# The Discovery of Potentially Selective Human Neuronal Nitric Oxide Synthase (nNOS) Inhibitors: A Combination of Pharmacophore Modelling, CoMFA, Virtual Screening and Molecular Docking Studies

**DOI:** 10.3390/ijms15058553

**Published:** 2014-05-14

**Authors:** Guanhong Xu, Yue Chen, Kun Shen, Xiuzhen Wang, Fei Li, Yan He

**Affiliations:** 1 School of Pharmacy, Nanjing Medical University, Nanjing 210029, China; E-Mails: xgh@njmu.edu.cn (G.X.); yuechen0311@126.com (Y.C.); mokingsk@sina.com (K.S.); showshow828@163.com (X.W.); 2 Department of Internal Neurology, Nanjing Children’s Hospital Affiliated to Nanjing Medical University, Nanjing 210008, China; 3 School of Pharmacy, China Pharmaceutical University, Nanjing 210009, China

**Keywords:** neuronal nitric oxide synthase, inhibitors, pharmacophore, virtual screening, docking

## Abstract

Neuronal nitric oxide synthase (nNOS) plays an important role in neurotransmission and smooth muscle relaxation. Selective inhibition of nNOS over its other isozymes is highly desirable for the treatment of neurodegenerative diseases to avoid undesirable effects. In this study, we present a workflow for the identification and prioritization of compounds as potentially selective human nNOS inhibitors. Three-dimensional pharmacophore models were constructed based on a set of known nNOS inhibitors. The pharmacophore models were evaluated by Pareto surface and CoMFA (Comparative Molecular Field Analysis) analyses. The best pharmacophore model, which included 7 pharmacophore features, was used as a search query in the SPECS database (SPECS^®^, Delft, The Netherlands). The hit compounds were further filtered by scoring and docking. Ten hits were identified as potential selective nNOS inhibitors.

## 1. Introduction

Nitric oxide (NO) is one of the most studied biological signaling molecules and is produced by catalysis from nitric oxide synthase (NOS), which converts l-arginine to l-citrulline, and produces this tiny, short-lived molecule. To date, there are three distinct isoforms of NOS: neuronal NOS (nNOS), endothelial NOS (eNOS) and inducible NOS (iNOS). nNOS and eNOS are constitutively expressed and depend on increases in external calcium and binding of a calcium/calmodulin complex for activation. nNOS and eNOS play an important role in neurotransmission and smooth muscle relaxation, respectively, and iNOS is expressed during bacterial infection, tumor cell cytolysis and inflammation [1–3].

As an inorganic reactive free radical gas, NO is believed to be involved in a number of physiological processes such as inflammation, neurotransmission, blood pressure regulation, platelet aggregation and pain [4–6]. However, overproduction of NO has been implicated in numerous disease states [7]. In particular, excess NO in the central nervous system from nNOS activity can lead to many neurological disease states including neurodegeneration during Alzheimer’s and Parkinson’s diseases [8], altered spinal transmission of neuropathic pain [9,10], and progression of migraine and chronic tension-type headaches [11]. Consequently, an inhibitor of nNOS has the potential to be therapeutic in these diseases; however, the functions of eNOS in blood pressure regulation and iNOS in immune responses must be preserved, and the selective inhibition of nNOS has been the challenge of many researchers in the past decade [12–15].

Recently, several categories of selective inhibitors of nNOS have been designed and developed for the treatment of central nervous system (CNS) disorders [3,15–22]. Some showed significant efficacy in the rat Chung model of neuropathic pain [22] and in a rodent model of dural inflammation relevant to migraine pain [22]. X-ray structures of nNOS co-crystallized with various ligands [23–25] provided insights into the essential structural elements and motifs central to its catalytic mechanism and mode of binding. These findings provide useful information about the interaction between the ligands and the residues near the binding site and can be utilized to design even more selective and potent drug-like NOS inhibitors.

Virtual screening based on a pharmacophore model as a 3D search query has been successfully employed as an efficient alternative to high throughput screening approaches for the development of new compounds with the desired biological properties [26]. Pharmacophore modeling can be used to analyze the common functional groups responsible for specific drug receptor interactions or as a prelude to three dimensional quantitative structure activity relationship (3D-QSAR) analyses that are aligned accordingly with a set of known active compounds in 3D space. 3D-QSAR has been successfully applied in drug discovery and design. As a popular QSAR method, Comparative Molecular Field Analysis (CoMFA) [27] studies incorporate 3D information of the ligands by searching for the sites on molecules that are capable of being modified into more specific ligands. As a useful methodology for studying interaction mechanisms, receptor based molecular docking analysis can be used as a complementary tool to prioritize the hits from the pharmacophore-based virtual screening [28].

In the present study, a 3D pharmacophore model for nNOS inhibitors was assembled and the generated model was used as a search query in the SPECS database containing 197,000 compounds. The virtual screening approach, in combination with pharmacophore modeling and molecular docking can be used to identify and design novel nNOS inhibitors with high selectivity. These molecules may be potential lead compounds for future drug development.

## 2. Results and Discussion

### 2.1. Pharmacophore Results

Twenty pharmacophore models were generated using SYBYL X 1.3 (Tripos Associates Inc., St. Louis, MO, USA). Table 1 lists the parameters of each model. Specificity is a logarithmic indicator of the expected discrimination for each query and is based on the number of features it contains, their allotment across partial match constraints, and the degree to which the features are separated in space. Strong models should have a high Specificity value. Generally, the Specificity value should be at least 5 in a pharmacophore model used as the query for a UNITY flex search [29]. For this study, MODEL 012, MODEL 019, and MODEL 003 had the high Specificity values of 5.138, 5.128 and 4.8580, respectively. These models yielded reasonable pharmacophore models. The N_HITS column shows the actual number of ligands hit by the model query, with the majority of the models matching at least 5 ligands. The value in the FEATS column indicates the total number of features possessed by each model. All of the models had six or more features except for MODEL 011. The retained models had a PARETO rank value of 0, indicating that a single model is not superior to any other. The HBOND term is a measure of the overall pharmacophoric similarity among the ligand conformers. The STERIC term is a measure of the overall steric similarity among the ligand conformers; this term is basically the same as the HBOND term. The ENERGY term indicates the total energy (using the Tripos force field) of all molecules in the training set.

**Figure 1.**
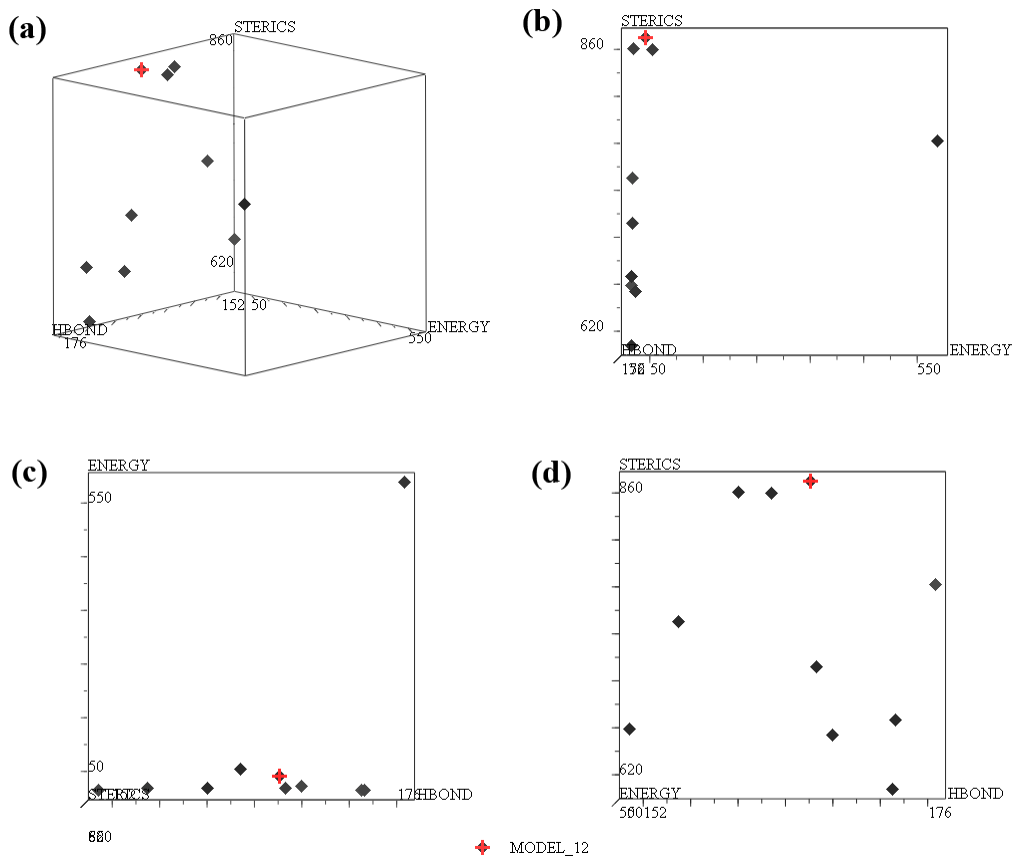
Plot of the STERICS, ENERGY and HBOND values for the models with the top ten Specificity values. (**a**) 3D plot; (**b**) plot of STERICS *vs.* ENERGY; (**c**) plot of ENERGY *vs.* HBOND; (**d**) plot of STERICS *vs.* HBOND. The red cross represents MODEL_12.

The most significant pharmacophore hypothesis was characterized by the conflicting demands of maximizing pharmacophore consensus, maximizing steric consensus, and minimizing conformer potential energy [30]. We constructed a 3D plot to visualize the Pareto surface and select the best pharmacophore model (Figure 1). Considering only the ENERGY and STERICS criteria, the best model is shown in the upper left-hand corner of the graph in Figure 1b, where the ENERGY is low and the STERICS score is high. In terms of ENERGY and HBOND criteria, the best model is shown in the lower part of the graph in Figure 1c, where the ENERGY is low and the HBOND score is high. Finally, in terms of ENERGY and HBOND, the best model is shown in the upper part of the graph, where both scores are high (Figure 1d). Among the considered models, MODEL_012 (represented with a red cross in Figure 1) has the optimal position because it fulfills the three criteria and it has the highest Specificity value [31].

**Figure 2.**
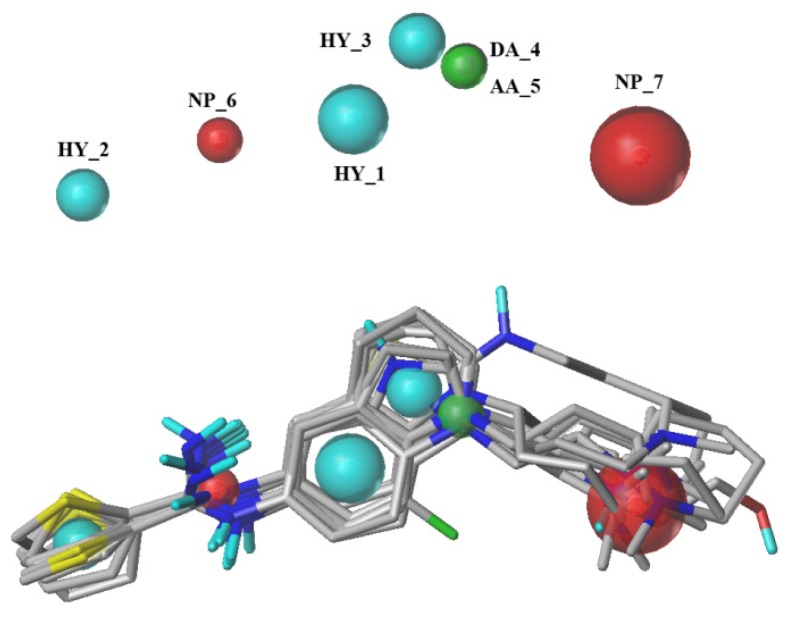
Selected pharmacophore MODEL_012 and the molecular alignment of the compounds used to elaborate the model.

The best GALAHAD MODEL 012 is displayed in Figure 2. All of the aligned conformers represent low-energy conformations of the molecules, and the final alignment shows a satisfactory superimposition of the pharmacophoric points. Cyan, magenta, green and red spheres indicate hydrophobes, donor atoms, acceptor atoms and positive nitrogens, respectively. Model 012 includes 7 pharmacophore features: three hydrophobes (HY_1, HY_2 and HY_3), one donor atom (DA_4), one acceptor atom (AA_5) and two positive nitrogens (NP_6 and NP_7). The magenta sphere is covered by a green sphere because the donor atom and the acceptor atom are in the same position in this molecule.

### 2.2. CoMFA (Comparative Molecular Field Analysis) Statistical Results

**Figure 3.**
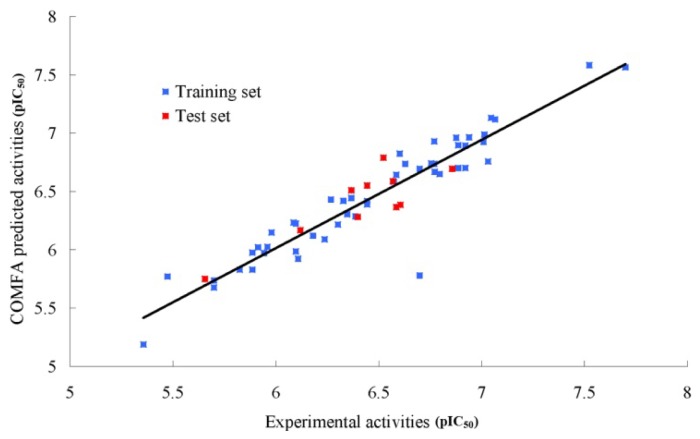
Correlation between the experimental and CoMFA (Comparative Molecular Field Analysis) predicted activities of compounds.

We used MODEL 012 as a template to align all molecules. The generated steric and electrostatic fields were scaled by the CoMFA-Standard scaling method in SYBYL with the default energy cutoff value. The CoMFA model yielded a good cross-validated correlation coefficient (*q*^2^) of 0.513 with an optimized component value of 4, which suggests that the model should be a useful tool for predicting the IC_50_ values. A high non-cross-validated correlation coefficient (*r*^2^_ncv_) of 0.933 with a low standard error estimate (SEE) of 0.134 and an *f* value of 149.950 were obtained. The steric and electrostatic contributions were 45.1% and 54.9%, respectively. The predicted activities for the inhibitors are listed in Table 2 and the correlation between the predicted activities and the experimental activities is depicted in Figure 3. The predictive correlation coefficient (*r*^2^_pred_) was 0.742 for the test set. The statistical results indicate that the CoMFA model is a reliable predictor.

**Figure 4.**
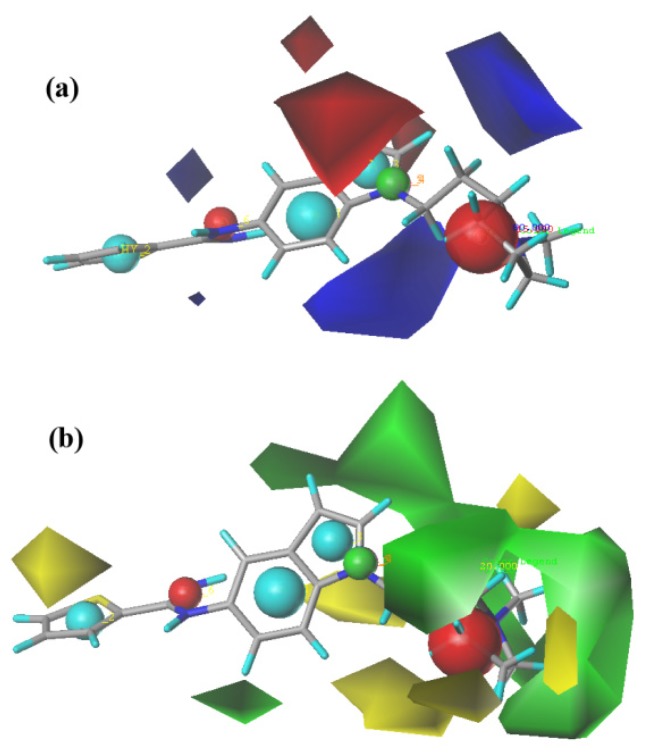
(**a**) CoMFA steric contour maps and (**b**) CoMFA electrostatic contour maps.

The CoMFA steric and electrostatic contour maps are shown in Figure 4 using compound 41 as a reference structure. In Figure 4a, the blue contour indicates regions in which an increase of positive charge enhances the activity, and the red contour indicates regions in which more negative charges are favorable for activity. The two large blue contours around the red sphere indicate that the substituent in this region should be electron deficient for increased binding affinity with a protein. Another small blue contour is found around the guanidine isosteric group indicating that a negatively charged substituent in this area is unfavorable. The CoMFA model showed the same result as the pharmacophore hypothesis. In Figure 4b, the steric field is represented by green and yellow contours, in which the green contours indicate regions where a bulky group is favorable and the yellow regions represent regions where a bulky group will decrease activity. In this case, the green contours around the substituent R demonstrated that bulky groups enhance the binding affinity of the nNOS. Most compounds with high activities in this dataset have the same such properties. The CoMFA contour maps and the predicted result further indicated that MODEL 012 can be used as a theoretical screening tool that is able to discriminate between active and inactive molecules [31].

### 2.3. Virtual Screening

**Figure 5.**
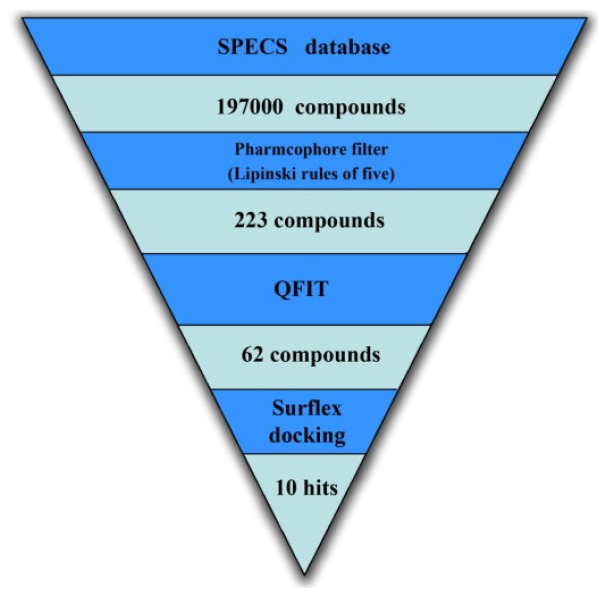
Virtual screening flowchart.

The pharmacophore based virtual screening was conducted to find potential nNOS inhibitors. A stepwise virtual screening procedure was applied, wherein the pharmacophore based virtual screening was followed by drug-likeness evaluation, screening of the pharmacophore query, QFIT (The QFIT score is a value between 0 and 100, where 100 is best and represents how close the ligand atoms match the query target coordinates within the range of a spatial constraint tolerance) scoring filtration, and a molecular docking study. The sequential virtual screening flowchart we employed is depicted in Figure 5, in which the reduction in the number of hits for each screening step is shown.

#### 2.3.1. Database Searching

Flexible 3D screening was performed using the UNITY tool to screen the SPECS database [32], which contains approximately 197,000 compounds. The database query was generated based on the pharmacophore MODEL 012. The database was restricted with Lipinski’s rule. In general, this rule describes molecules that have drug-like properties. Drug-likeness is a property that is most often used to characterize compound libraries such as combinatorial or screening libraries that are screened to find novel lead chemical compounds [33]. According to this rule, we used simple molecular descriptors, such as molecular weight (≤500), hydrophobicity (MLogP ≤ 4.15) and the number of H-bond donor (≤5) and acceptor atoms (≤10), as the first filter to select the molecules with good absorption or permeation [34]. The remaining 223 compounds were further screened on the basis of QFIT to reduce the dataset, where QFIT is the pharmacophore match between the query and hit [35].

#### 2.3.2. Molecular Docking

**Figure 6.**
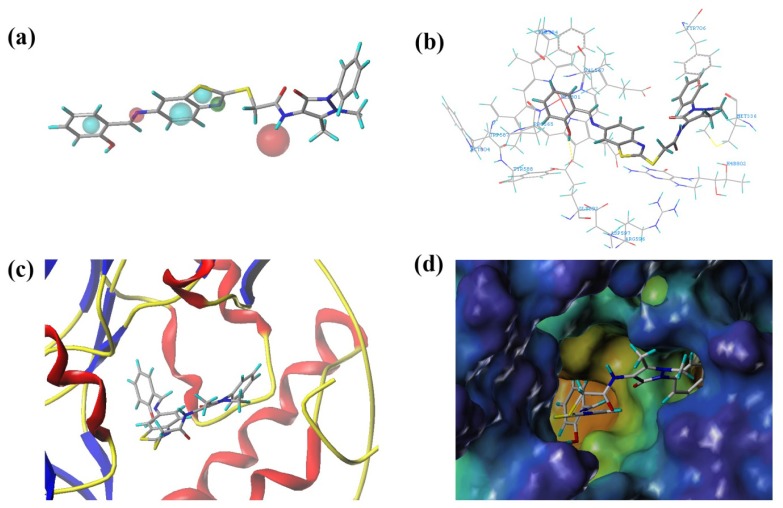
(**a**) Mapping of the hit molecule (AG_205/36953325) by MODEL 012 from SPECS databases; (**b**) The orientation of AG_205/36953325 in the active site of nNOS; (**c**) The secondary structure of the active site and AG_205/36953325; and (**d**) The MOLCAD (a software package of SYBYL) cavity depth potential surfaces structure of the binding site within AG_205/36953325. The cavity depth color ramp ranges from blue (outside of the pocket) to light red (cavities deep inside the pocket).

To predict the appropriate binding conformation for nNOS inhibitors and the reported hit compounds from virtual screening, Surflex Docking (Tripos Associates Inc., St. Louis, MO, USA) was used to generate an ensemble of docking conformations. The top 62 hit compounds with the highest QFIT score from screening after UNITY filtering were further screened using molecular docking into the binding site of nNOS to select the compounds with the ability to form favorable interactions with the active site. The docked compounds were filtered based on scoring function and interaction with the crucial residues [24] in the binding site. Finally, ten compounds were selected on the basis of the dock score and favorable interactions with the key residues. The results of the hit compounds with their dock score and QFIT values are shown in Table 3. Among the active compounds reported [24], AG_205/36953325 has a similar linker length and two aromatic ring centers on both ends. There is also a hydrogen bond donor in the aromatic ring center and at least one hydrogen bond donor on the linker. The phenolic hydroxyl makes a hydrogen bond with the NOS active site GLU592, which is conserved in all mammalian NOS isoforms [24], and the hydrophobic phenyl ring π-stacks with the heme (HEM801) next to the GLU. The long, flexible linker extending from the phenyl ring allows the 2-phenyl-2,3-dihydro-1H-pyrazole to reach and to π-stack with TYR706 (Figure 6). The binding mode of this hit compound is similar to that of the reported co-crystallized compound [24] and indicates that the identified hit compounds may have the same mechanism of action as known nNOS inhibitors.

## 3. Experimental Section

### 3.1. Compounds and Biological Data

Fifty-nine novel nNOS inhibitors were taken from the literature [15–17,21,22] with their biological activities in terms of IC_50_ values; 49 compounds were used as a training set and the remaining 10 compounds were used as a test set, based on random selection. The compounds in the test set have a range of biological activity values similar to that of the training set. The IC_50_ values of the inhibitors were converted into pIC_50_ (log (1/IC_50_)) and used as dependent variables in the Pharmacophore generation and CoMFA calculations. The structures of the compounds and their pIC_50_ values are given in Table 2. All molecular modeling calculations were conducted using SYBYL X 1.3 (Tripos Associates Inc.). Molecular building was performed with a molecule sketch program in the same software. The molecular geometry of each compound was first minimized using the standard Tripos molecular mechanics force field with 0.01 kcal/(mol A) energy gradient convergence criterion. Partial atomic charges were calculated by the Gasteiger-Hückel method and energy minimizations were performed using the Conjugate Gradient method with 1000 iterations [36,37].

### 3.2. Pharmacophore Generation

Pharmacophore models were generated and analyzed using the GALAHAD module. In this study, ten compounds (13, 16, 19, 21, 24, 30, 40, 41, 43, and 52) were selected to carry out the pharmacophore hypothesis, and the genetic algorithm was used to create conformers for all molecules. The compounds that were selected to generate the pharmacophore hypothesis are highly active. All of the ligands were aligned with a population size value of 60, a maximum generation value of 60 and a value of molecular required hitting of 5. Twenty models were generated with default parameters.

### 3.3. CoMFA Field Calculation Partial Least Square Analysis

The standard CoMFA procedure as implemented by SYBYL X 1.3 (Tripos Associates Inc.) was performed. Each set of aligned molecules was positioned inside a 3D cubic lattice with a grid spacing of 2.0 A (default distance) in all Cartesian directions and was generated to enclose the molecule aggregate. The fields generated were automatically scaled by the CoMFA standard in SYBYL. The partial least squares (PLS) methodology was used to derive a linear relationship for the CoMFA, and cross-validation was performed using the leave-one-out (LOO) method to choose the optimum number of components (ONC) and assess the statistical significance of each model. In PLS, the independent variables were the CoMFA descriptors, and the pIC_50_ values were used as dependent variables. The ONC was the number of components that led to the highest cross-validated correlated correlation coefficient *q*^2^ (or *r*^2^_cv_). Non-cross-validation was performed to calculate conventional *r*^2^_ncv_ using the same number of components [38–40].

### 3.4. Virtual Screening

The selected pharmacophore model was validated and converted into a UNITY query for pharmacophore guided virtual screening studies. The query was screened against the SPECS database. The flex search option was implemented to perform virtual screening. Primary filters such as Lipinski’s rule of five were applied to reduce the dataset. Further screening of the hits was carried out using the Surflex Dock in SYBYL.

The docking study was performed to validate the hits obtained from the virtual screening. The crystal structure of nNOS was retrieved from the RCSB Protein Data Bank (PDB code: 4EUX) [24]. The nNOS structure was utilized in subsequent docking experiments without energy minimization. Protein structures were prepared using the biopolymer module of SYBYL. Hydrogen atoms were added to the structure, atom types and charges were assigned using AMBER7 FF99 force field, and side chain amides were modified. The ligand method was used as the mode of construction for the protomol, threshold and bloat using default values to determine the extent of the protomol.

## 4. Conclusions

nNOS is a therapeutic target for central nervous system diseases that has attracted interest from pharmaceutical companies and researchers. Selective inhibition of nNOS activity represents an exciting drug approach for the development of new therapeutic agents to treat neurodegenerative diseases. In this study, we described a rational strategy for identifying novel nNOS inhibitors using a pharmacophore-based virtual screening protocol. The best pharmacophore model (MODEL 012) was established and showed good statistical parameters in the validation process. MODEL 012 was further employed as a 3D search query to screen the SPECS compound database. Molecular docking studies were also performed to improve the reliability and accuracy of the virtual screening. Ten hit compounds were identified as potential selective nNOS inhibitors and exhibited good search scoring, high docking scores, similar binding mode to experimentally proven compounds and favorable drug-like properties. The pharmacophore models developed in this work, and the information gained about the interactions between nNOS and the potential selective inhibitors, indicated that the combination of pharmacophore, molecular docking, and virtual screening efforts is a successful approach for identifying effective inhibitory compounds that may have an impact on future experimental studies in selective nNOS inhibition. The identified hit compounds were structurally different from available inhibitors and may serve as potential leads or starting points for structural optimization to identify novel nNOS inhibitors.

## Figures and Tables

**Table 1.** Parameters of the pharmacophore model a.

No.	SPECIFICITY	N_HITS	FEATS	PARETO	ENERGY	STERICS	HBOND
MOEDL_001	4.180	4	6	0	15.60	666.7	173.3
MOEDL_002	3.881	8	7	0	15.44	703.1	161.8
MOEDL_003	4.858	6	8	0	18.53	750.4	155.1
MOEDL_004	4.108	6	6	0	18.62	712.0	166.6
MOEDL_005	3.823	9	7	0	20.15	852.7	162.7
MOEDL_006	3.735	6	7	0	17.54	714.7	160.5
MOEDL_007	3.902	9	7	0	58.38	705.2	179.2
MOEDL_008	4.051	6	6	0	19.3	784.6	171.4
MOEDL_009	4.036	3	6	0	40.81	845.3	159.5
MOEDL_010	3.393	5	9	0	35.12	612.2	178.2
MOEDL_011	3.158	6	5	0	22.40	635.3	178.9
**MOEDL_012** b	**5.138**	**5**	**7**	**0**	**41.13**	**870.1**	**166.1**
MOEDL_013	4.048	6	6	0	17.75	732.8	157.0
MOEDL_014	4.124	6	6	0	19.25	861.2	160.2
MOEDL_015	3.867	8	7	0	19.99	567.7	175.3
MOEDL_016	4.050	5	6	0	23.76	834.0	165.7
MOEDL_017	4.053	5	6	0	14.97	658.9	150.8
MOEDL_018	4.058	5	6	0	55.07	859.6	162.8
MOEDL_019	5.128	4	7	0	23.06	654.1	168.0
MOEDL_020	4.050	5	6	0	16.91	748.1	158.3

a SPECIFICITY is a logarithmic indicator of the expected discrimination for each query; N_HITS is the actual number of ligands hit by the model query; FEATS is the total number of features in the model query; PARETO indicates the Pareto rank of the each model; ENERGY is the total energy of the model; STERICS is the steric overlap for the model; HBOND is the pharmacophoric concordance;

b The selected model (MODEL_012) is indicated in boldface.

**Table 2.** Structure and biological values (pIC_50_) of nNOS inhibitors.

No.		Structure	pIC_50_	

			Observed	Predicted
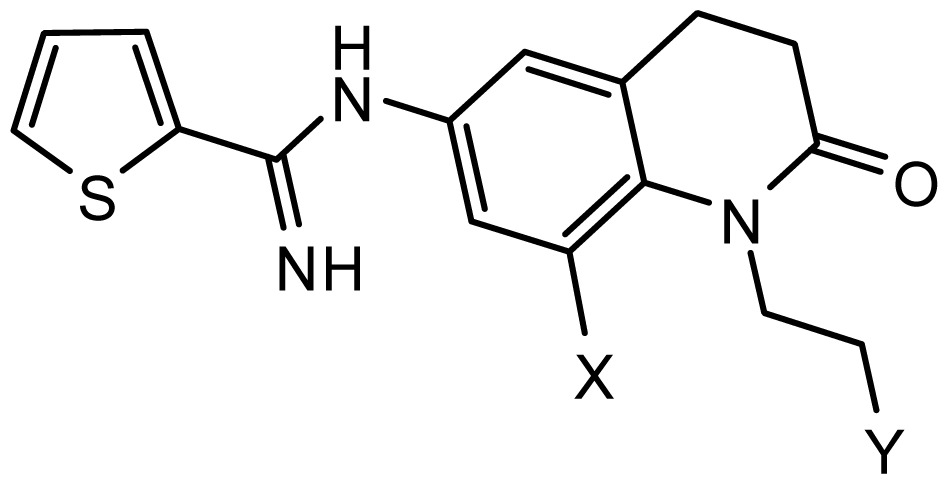 *series 1* [22]				

-	X	Y	-	-

1	H	N(CH_3_)_2_	6.237	6.089
2 *	H	N(Et)_2_	5.656	5.750
3	H	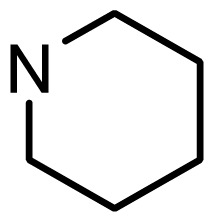	6.108	5.922
4	H	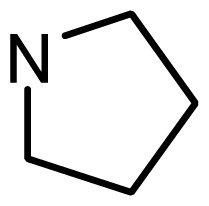	6.796	6.650
5	H	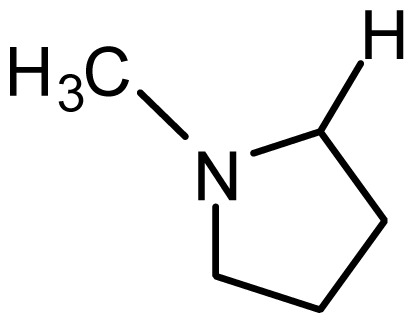	5.979	6.148
6	F	N(CH_3_)_2_	5.474	5.770
7	H	CH_2_N(CH_3_)_2_	5.943	5.971
8	H	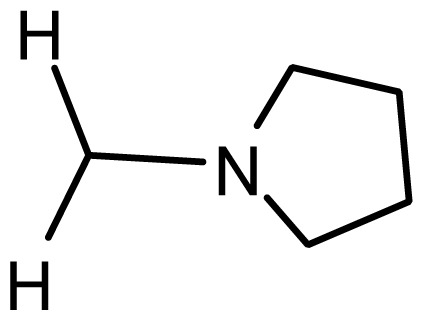	5.914	6.021

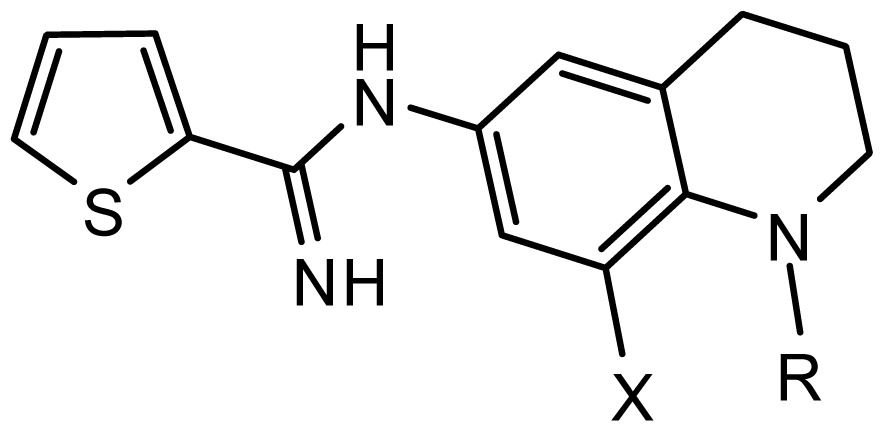 *series 2* [15,22]				

-	X	R	-	-

9 *	H	−CH_2_CH_2_CH_2_N(CH_3_)_2_	6.569	6.588
10	H	−CH_2_CH_2_NCH_3_	6.754	6.741
11 *	H	−CH_2_CH_2_N CH_2_CH_3_	6.857	6.694
12	H	−CH_2_CH_2_NCH(CH_3_)_2_	6.573	6.585
13	H	−CH_2_CH_2_N(CH_3_) (C_2_H_5_)	7.013	6.987
14 *	H	−CH_2_CH_2_N(CH_3_)_2_	6.367	6.510
15	H	−CH_2_CH_2_N(C_2_H_5_)_2_	6.585	6.642
16	F	−CH_2_CH_2_N(C_2_H_5_)_2_	7.032	6.757
17	H	− (CH_2_)_3_NCH_3_	6.629	6.736
18	H	−CH_2_CH_2_N(CH_3_) (CH_2_)_2_OH	6.876	6.960
19	H	− (CH_2_)_2_NH(CH_2_)_2_OH	6.939	6.964
20	H	− (CH_2_)_3_NH(CH_2_)_2_OH	6.772	6.667
21	H	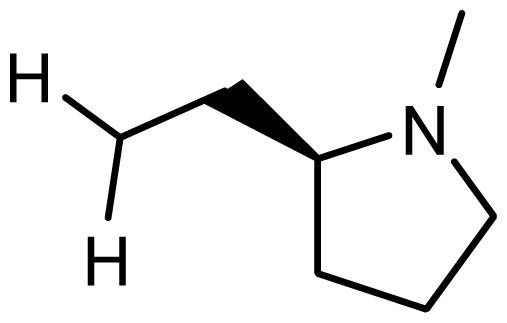	7.009	6.925
22	H	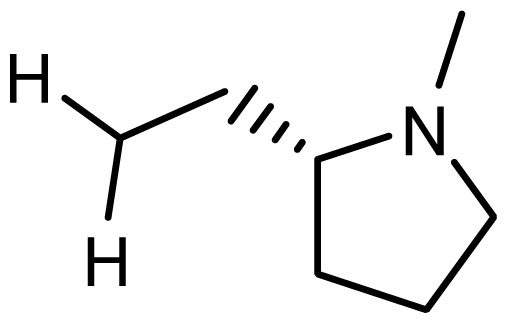	6.886	6.896
23 *	H	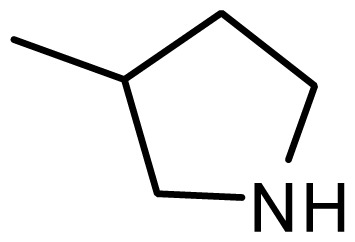	6.606	6.385
24	H	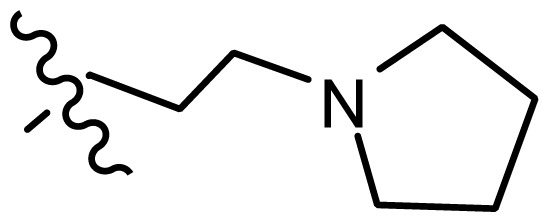	7.066	7.118
25	H	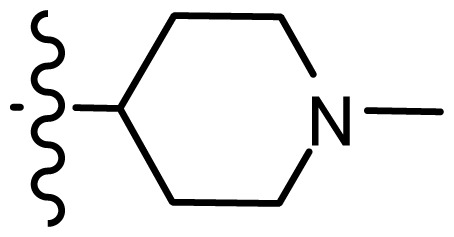	6.086	6.233
26	H	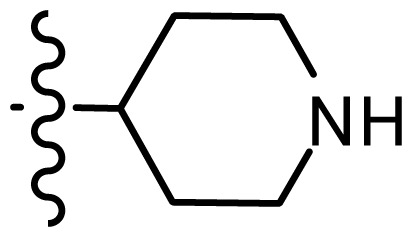	6.268	6.430
27 *	H	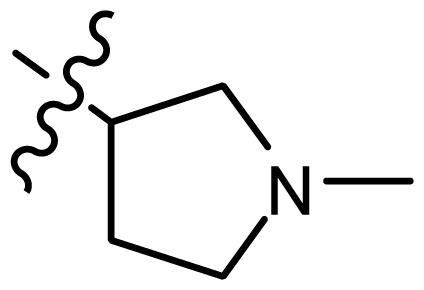	6.444	6.550

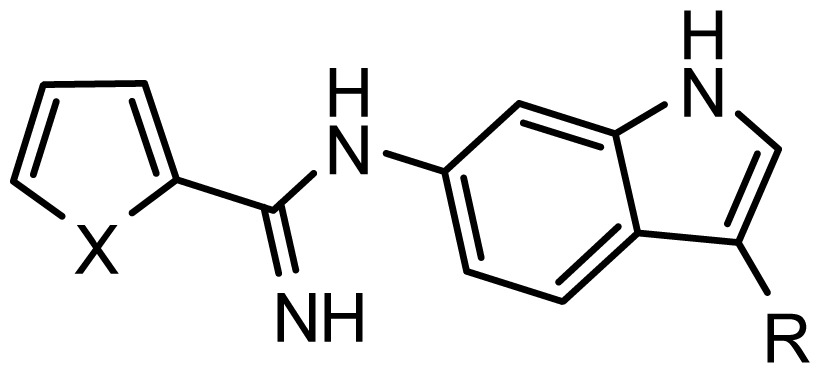 *series 3* [21]				

-	X	R	-	-

28	S	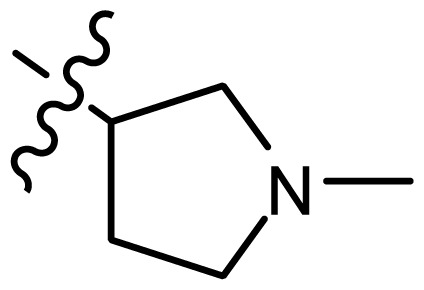	6.699	6.694
29	S	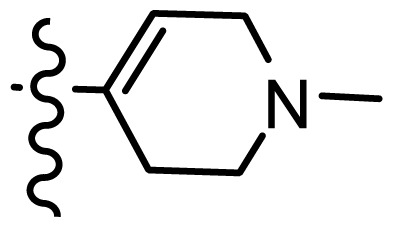	6.097	6.225
30	S	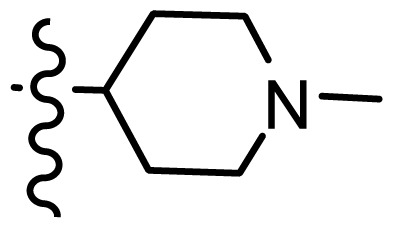	6.921	6.701
31	S	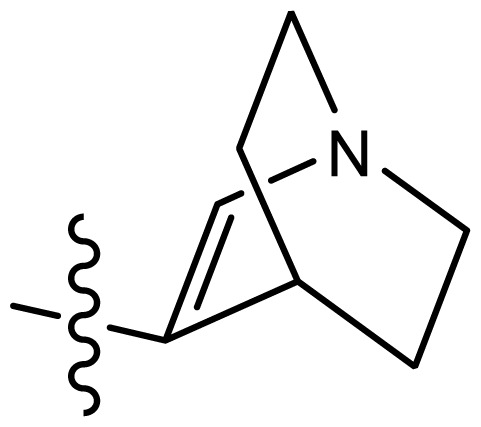	5.824	5.830
32	S	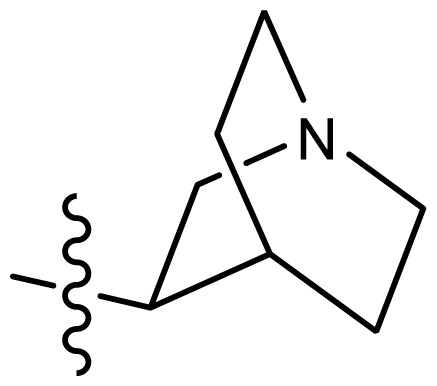	6.347	6.304

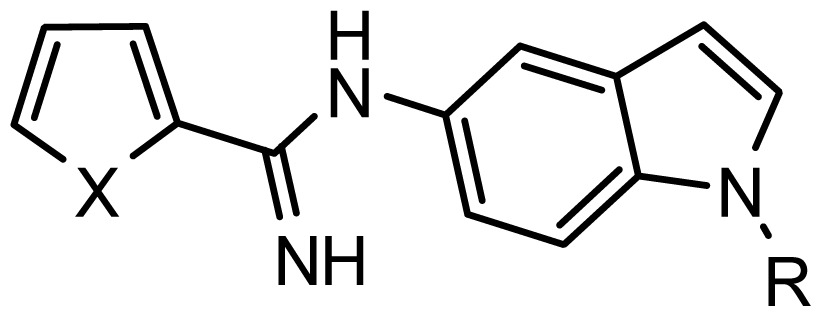 *series 4* [17]				

-	X	R	-	-

33	S	*N*-(1-(3-(dimethylamino)propyl)-	6.328	6.419
34 *	S	*N*-(1-(3-(cyclopropylamino)propyl)-	6.585	6.366
35	S	*N*-(1-(3-morpholinopropyl)-	6.181	6.120
36	S	*N*-(1-(3-((1-ethylpyrrolidin-2-yl)methylamino)propyl)-	6.886	6.700
37	S	*N*-(1-(3-adamantanaminopropyl)-	6.444	6.388
38	S	*N*-(1-(2-(dimethylamino)ethyl)-	6.770	6.736
39	S	*N*-(1-(2-(piperidin-1-yl)ethyl)-	6.770	6.930
40	S	*N*-(1-(2-(1-methylpiperidin-2-yl)ethyl)-	7.046	7.131
41	S	(*S*) *N*-(1-(2-(1-methylpyrrolidin-2-yl)ethyl)-	7.700	7.564
42	O	*N*-(1-(2-(1-methylpyrrolidin-2-yl)ethyl)-	6.602	6.824
43	S	*N*-(1-(1-methylazepan-4-yl)-	6.921	6.893
44	O	*N*-(1-(1-methylazepan-4-yl)-	6.367	6.443
45 *	S	*N*-(1-(8-methyl-8-azabicyclo[3.2.1]octan-3-yl)-	6.120	6.168
46	S	*N*-(1-(quinuclidin-3-yl)-	6.444	6.417
47	S	*N*-(1-(1-methylpiperidin-4-yl)-	6.387	6.286

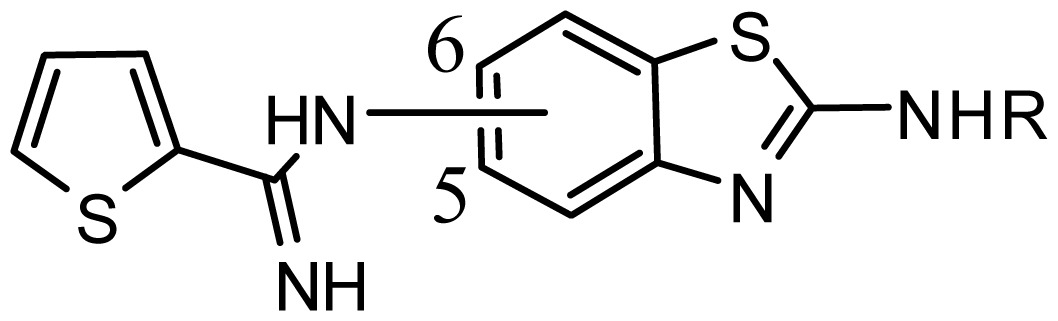 *series 5* [16]				

	Substituted	R		

48	5	2-(Pyridin-2-yl)ethyl	5.959	6.025
49	5	2-Morpholinoethyl	5.886	5.976
50 *	5	1-Benzylpiperidin-4-yl	6.398	6.281
51	5	1-(4-Fluorobenzyl)piperidin-4-yl	6.097	5.986
52	5	(±)-2-(1-Methylpyrrolidin-2-yl)ethyl	7.523	7.582
53	6	2-(Pyridin-2-yl)ethyl	5.886	5.83
54	6	2-Morpholinoethyl	5.699	5.676
55	6	1-Benzylpiperidin-4-yl	6.301	6.216
56	6	1-(4-Fluorobenzyl)piperidin-4-yl	6.699	5.779
57 *	6	2-(1H-Imidazol-5-yl)ethyl	6.523	6.789
58	6	4-Bromophenethyl	5.357	5.188
59	6	Tetrahydro-2H-pyran-4-yl	5.699	5.736

* Compounds taken for the test set.

**Table 3.** Chemical structures of the hit compounds and their dock scores and QFIT values.

SPECS ID	Structure	Dock Scores	QFIT
AG_205/36953325	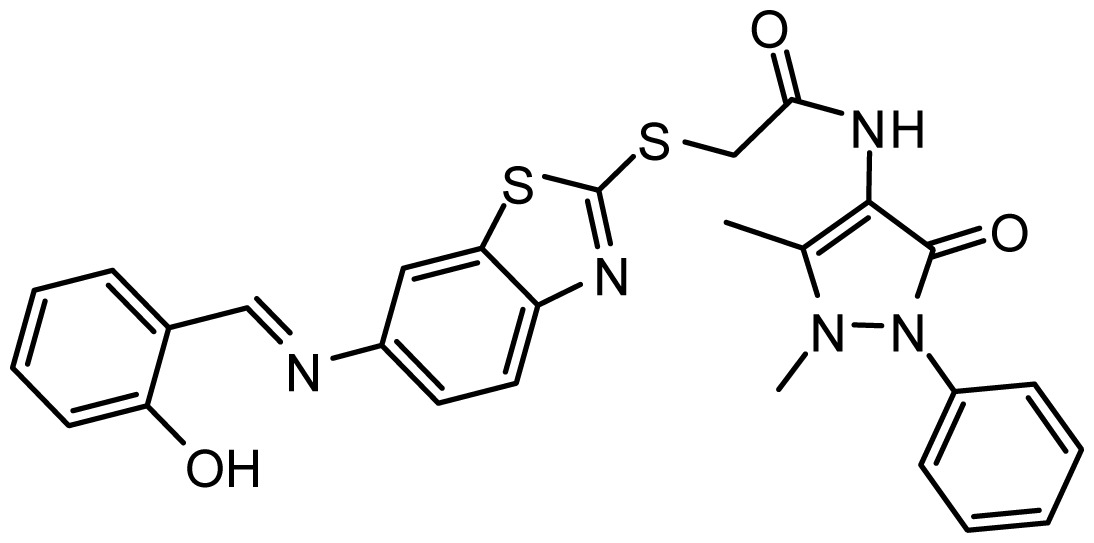	8.29	65.74
AG_205/11218159	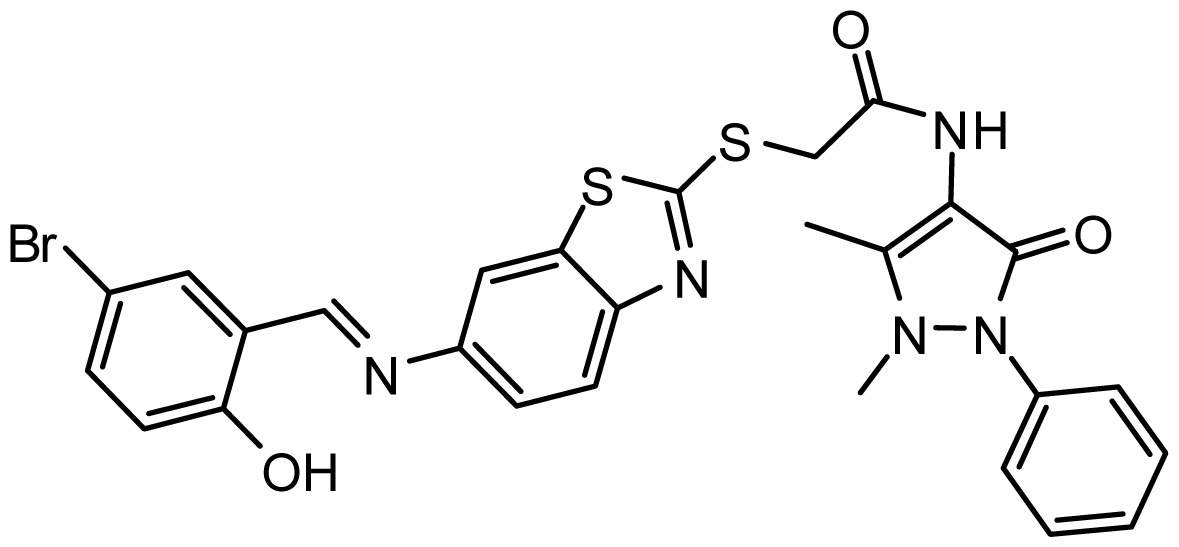	8.20	65.74
AG_205/11218337	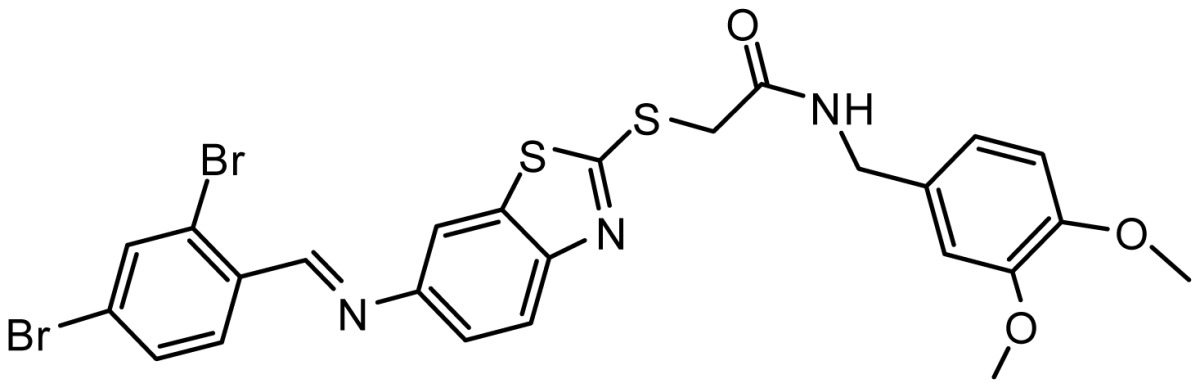	8.01	65.82
AG_205/11218321	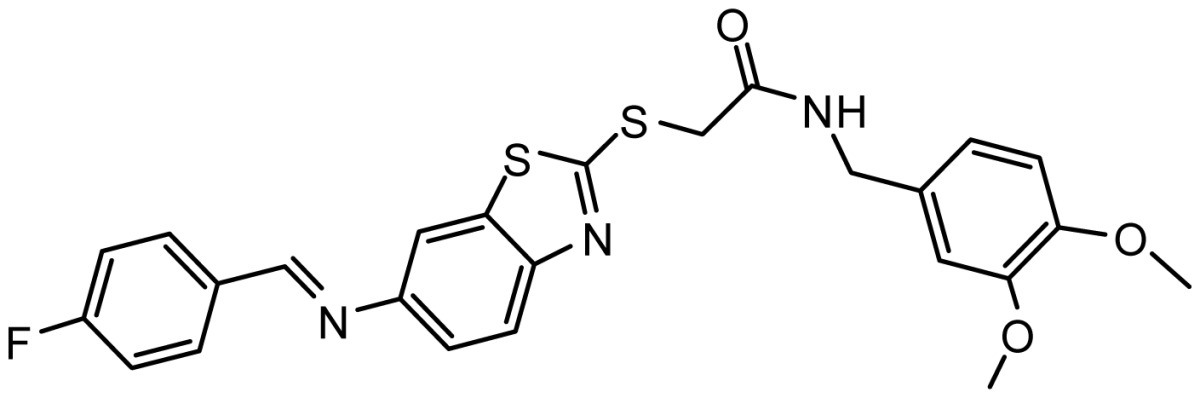	7.86	65.82
AG_205/36564022	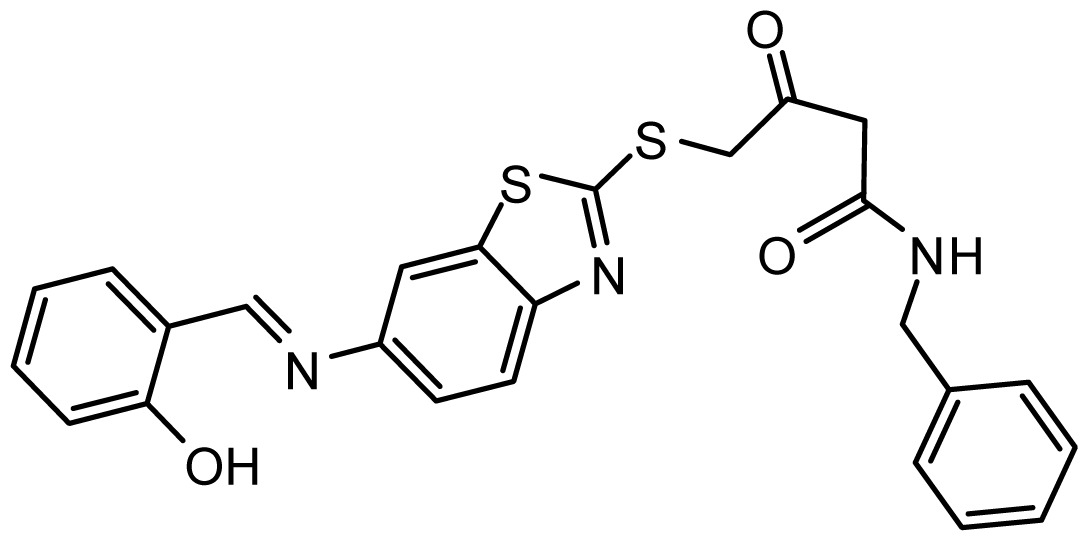	7.65	65.82
AG_205/36953138	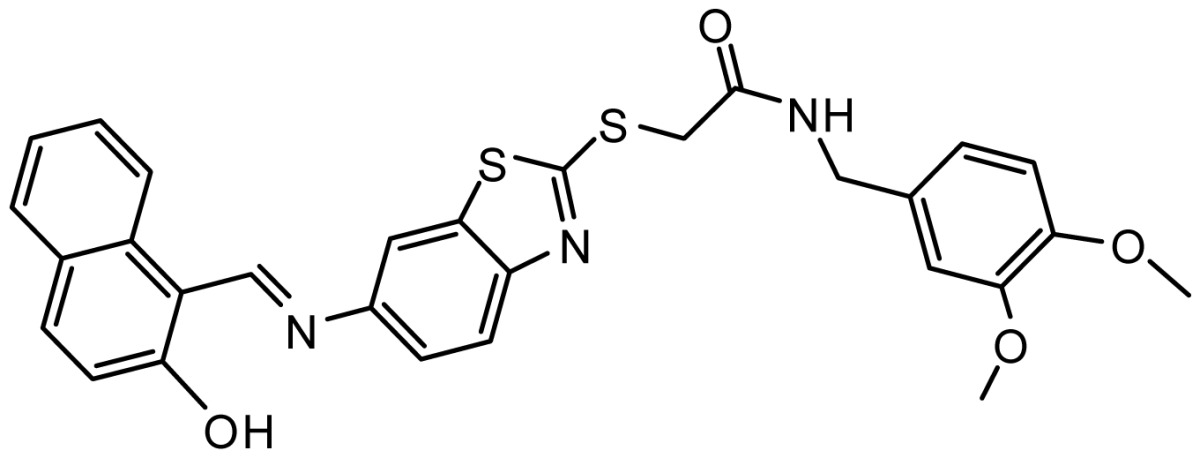	7.63	65.81
AG_205/09949027	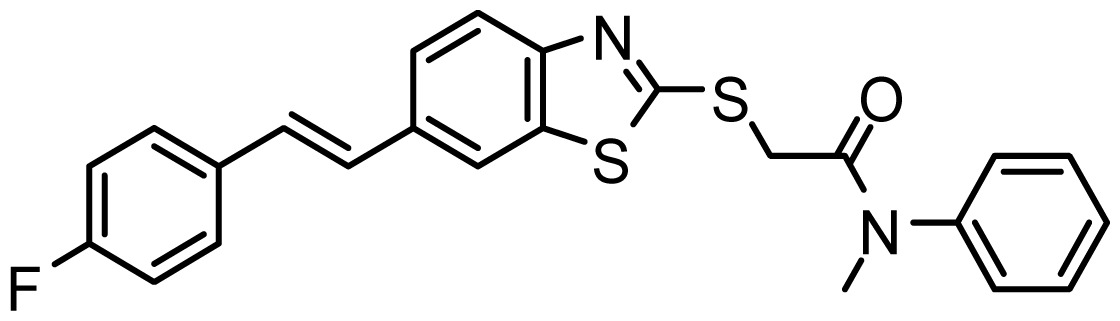	7.34	65.82
AG_205/36953406	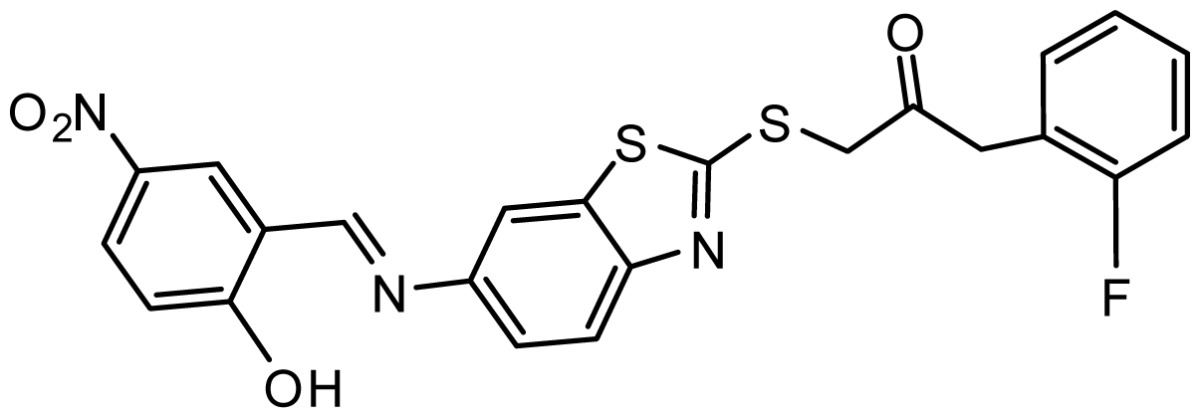	6.78	65.82
AG_205/36265063	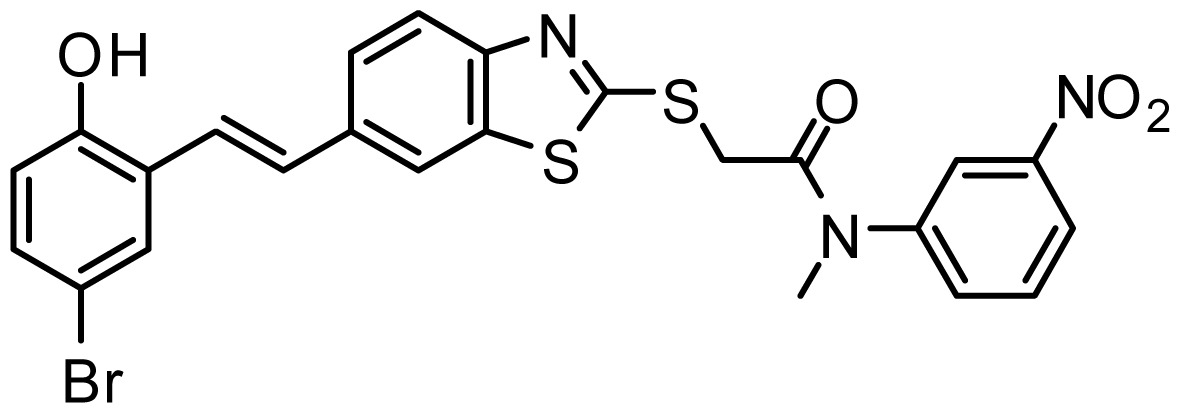	6.51	65.82
AG_205/36940042	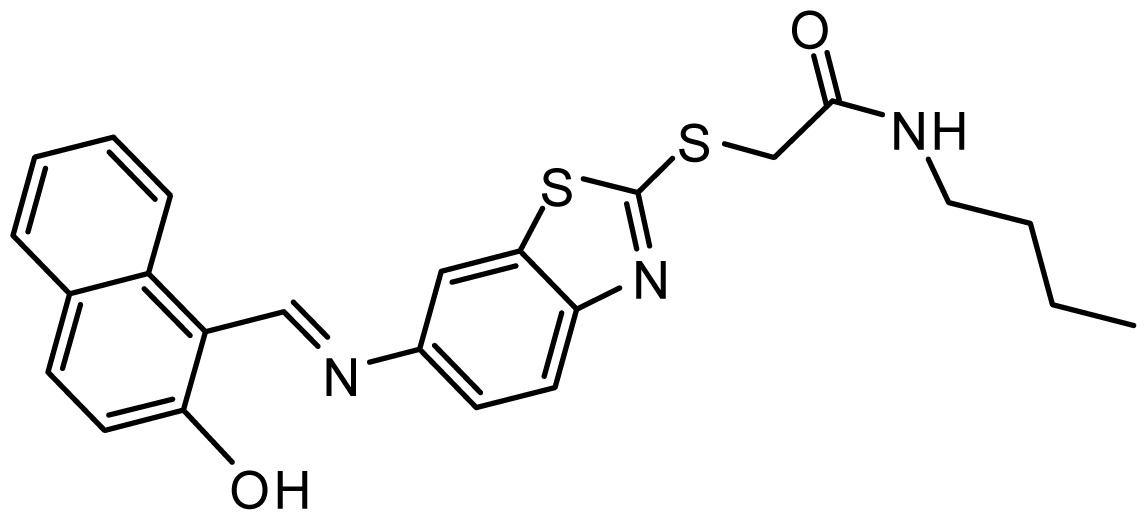	6.22	65.81
